# Computational Modeling and Characterization of Peptides Derived from Nanobody Complementary-Determining Region 2 (CDR2) Targeting Active-State Conformation of the β_2_-Adrenergic Receptor (β_2_AR)

**DOI:** 10.3390/biom14040423

**Published:** 2024-03-30

**Authors:** Milan Sencanski, Sanja Glisic, Valentina Kubale, Marko Cotman, Janez Mavri, Milka Vrecl

**Affiliations:** 1 Laboratory for Plant Molecular Biology, Institute of Molecular Genetics and Genetic Engineering, University of Belgrade, 11000 Belgrade, Serbia; 2 Laboratory for Bioinformatics and Computational Chemistry, Institute of Nuclear Sciences VINCA, National Institute of Serbia, University of Belgrade, 11000 Belgrade, Serbia; sanja@vinca.rs; 3 Institute of Preclinical Sciences, Veterinary Faculty, University of Ljubljana, 1000 Ljubljana, Slovenia; valentina.kubaledvojmoc@vf.uni-lj.si (V.K.); marko.cotman@vf.uni-lj.si (M.C.); 4 Department of Computational Biochemistry and Drug Design, National Institute of Chemistry, 1000 Ljubljana, Slovenia; janez.mavri@ki.si

**Keywords:** bioinformatics, nanobody-derived peptides, complementary-determining region 2, molecular modeling, β2-adrenergic receptor, cell-based in vitro assays

## Abstract

This study assessed the suitability of the complementarity-determining region 2 (CDR2) of the nanobody (Nb) as a template for the derivation of nanobody-derived peptides (NDPs) targeting active-state β_2_-adrenergic receptor (β_2_AR) conformation. Sequences of conformationally selective Nbs favoring the agonist-occupied β_2_AR were initially analyzed by the informational spectrum method (ISM). The derived NDPs in complex with β_2_AR were subjected to protein–peptide docking, molecular dynamics (MD) simulations, and metadynamics-based free-energy binding calculations. Computational analyses identified a 25-amino-acid-long CDR2-NDP of Nb71, designated P4, which exhibited the following binding free-energy for the formation of the β_2_AR:P4 complex (ΔG = −6.8 ± 0.8 kcal/mol or a Ki = 16.5 μM at 310 K) and mapped the β_2_AR:P4 amino acid interaction network. In vitro characterization showed that P4 (i) can cross the plasma membrane, (ii) reduces the maximum isoproterenol-induced cAMP level by approximately 40% and the isoproterenol potency by up to 20-fold at micromolar concentration, (iii) has a very low affinity to interact with unstimulated β_2_AR in the cAMP assay, and (iv) cannot reduce the efficacy and potency of the isoproterenol-mediated β_2_AR/β-arrestin-2 interaction in the BRET^2^-based recruitment assay. In summary, the CDR2-NDP, P4, binds preferentially to agonist-activated β_2_AR and disrupts Gαs-mediated signaling.

## 1. Introduction

Camelid-derived nanobodies (Nbs) are single-domain, recombinant, small, antigen-binding fragments of camelid heavy-chain-only antibodies with a wide range of research and potential therapeutic applications [1]. Nbs provides several advantages over classical antibodies, including smaller size, high stability, production yield in various expression systems, the ability to cross the plasma membrane, solubility, and the ability to recognize epitopes that traditional antibodies cannot access [2]. Nbs bind to specific antigens through their three complementarity-determining regions (CDR1, CDR2, and CDR3), relying mainly on CDR3 but also on CDR1 and CDR2 [2]. The high diversity within the CDR3 region, present across all antigen receptors, has been assumed to be a critical factor in the specificity of antigen recognition compared with the more cross-reactive CDR1 and CDR2 regions [3]. However, some studies have also highlighted the critical involvement of CDR2 in antibody–antigen interactions by showing that (i) mutations within CDR2 can significantly impair the antibody-binding capacity [4] and (ii) the binding affinity and toxin-neutralizing activity of Nb against ricin toxin depend on elements within CDR2 [5], indicating the potential of CDR2 for fine-tuning antigen recognition.

In the G protein-coupled receptors (GPCRs), one of the largest, highly diverse, and therapeutically relevant groups of membrane receptors, the use of Nbs has played a crucial role in achieving important breakthroughs. These include resolving the crystal structures of β_2_AR in complex with agonist/Gs [6,7,8]. As highlighted in various reviews [9,10,11,12,13], this methodology has also proved effective in resolving the active-state crystal structures of other GPCRs for imaging and modulating GPCR function and as a new class of potential GPCR-targeted therapeutics.

β_2_AR-specific intracellular binding nanobodies, termed “intrabodies,” have been developed that maintain their conformational specificity when expressed in cells [14]. Several intrabodies targeting the intracellular epitopes of different GPCRs have been developed, and an overview of their pharmacological activity and applications has been summarized by Heukers et al. [9]. Their unique convex shape enables them to bind between the intracellular loops of GPCRs and thus stabilize their conformation, which has helped elucidate the dynamic structural features of GPCRs (see [12]). As shown for the β_2_AR, the CDR3 amino acid sequence of Nb80 enters a hydrophobic pocket formed by amino acids of β_2_AR transmembrane segments 3, 5, 6, and 7, whereas the amino acids within CDR1 only stabilize this interaction [6].

Further advancements include the development of peptidomimetics, such as Nb-derived peptide mimetics (NDPs) and, more specifically, CDR-derived peptide mimetics [15]. However, the design and development of NDPs face challenges such as low binding affinity and biological activity. Studies have developed CDR3-derived peptidomimetics directed against β_2_AR [16,17] and the vascular endothelial growth factor [18]. In contrast to CDR3-derived peptidomimetics directed against β_2_AR, those directed against the vascular endothelial growth factor displayed binding energies similar to that of a full-length nanobody. Because the involvement of CDR2 is not as established, this study aimed to investigate the suitability of the CDR2 of conformationally selective Nbs favoring the agonist-occupied β_2_AR conformation as a template for the derivation of NDPs targeting the active conformation of the β_2_AR. This conformation was assessed using computational and experimental in vitro approaches, including (i) the virtual informational spectrum method (ISM) [19,20], (ii) membrane molecular dynamics (MD) and metadynamics simulations [21,22], and (iii) in vitro characterization to test the ability of computationally designed CDR2-NDP to cross the plasma membrane and interfere with agonist-induced β_2_AR Gαs-mediated signaling and β-arrestin 2 interaction. Compared with CDR3-derived peptide mimetics, CDR2-NDP P4 had similar binding affinities and the ability to impair agonist-induced β_2_AR Gαs-mediated signaling.

## 2. Materials and Methods

### 2.1. Material

Sigma-Aldrich (St. Louis, MO, USA) and the Gibco Invitrogen Corporation (Breda, The Netherlands) supplied the molecular biology reagents, cell culture media, and isoproterenol. CDR2-NDP (P4) and fluorescein isothiocyanate (FITC)-labeled P4 (FITC-P4) were custom synthesized (Biomatik Corporation; Cambridge, ON, Canada). CellMask™ Orange plasma membrane stain and Coelenterazine 400a were obtained from Thermo Fisher Scientific (Waltham, MA, USA) and Biotrend Chemikalien GmbH (Köln, Germany), respectively.

### 2.2. Informational Spectrum Method (ISM) and Computational Peptide Scanning

The ISM principle, described in previous studies [20,23], has been shown to be successful in analyzing protein structure–function [20] and predicting novel interactors [24], including GPCR interaction partners [25,26]. This method assigns a parameter to each amino acid based on the electron–ion interaction potential (EIIP). The ISM algorithm includes three steps: (i) converting amino acid sequences into numerical sequences using EIIP, (ii) converting the numerical sequence into an information spectrum (IS) through Fourier transformation, and (iii) performing cross-spectral analysis (CIS) to identify common biological properties or interactions. The effectiveness of the method was evaluated by peak analysis in the consensus information spectrum (CIS), where the most prominent peak indicated common frequency components among the analyzed proteins. The signal-to-noise ratio (S/N) is a measure of the similarity of individual peaks, with lower S/N values indicating lower interaction affinities between the proteins at specific frequencies.

### 2.3. Datasets and Computational Peptide Scanning

The sequence of human β_2_AR for bioinformatic analysis was obtained from UniProt (accession number P07550). The Nb sequences can be found in the US patent, US20130137856, and the PDB entry 3P0G FASTA sequence. Peptide scanning was used to identify the linear protein regions responsible for specific interaction(s) exhibiting the highest amplitudes at the predefined Fourier frequency.

### 2.4. Receptor Preparation

The crystal structures of β_2_AR in the active state (PDB entry 3P0G; [6]) and in the complex with Gs (PDB 3SN6; [7]) were retrieved from the RCSB Protein Data Bank. After the removal of the lipids, water molecules, ions, and Nb80, only the P0G ligand remained.

### 2.5. Molecular Dynamics Simulations

Details of the protocol involving molecular docking of peptides, ligand parameterization, molecular dynamics, and metadynamics simulations were described in a previous study [17]. The molecular dynamics simulation of peptide binding to the receptor site is substantially more demanding than that of rigid ligands, as the peptide moiety is flexible, and such degrees of freedom have long correlation times. Long simulation times are necessary to reproduce the binding free energies of these flexible ligands. The CABS-dock server was used for peptide to protein docking [27]. The simulation included 50 cycles, and the solutions that resulted in intracellular peptide placement and the lowest CABS docking energy were selected for subsequent MD simulations. The complex derived from the docking results (agonist-bound β_2_AR:P4) was placed in a 70 × 70 Å 2-oleoyl-1-palmitoyl-sn-glyecro-3-phosphocholine (POPC) lipid bilayer. A 10 Å-thick water layer was added, and water molecules with poor contact were removed. Neutralization with 0.15 M NaCl resulted in a 61,183 (~60,000)-atom ensemble. The system was then subjected to energy minimization, followed by 250 NVE ps of equilibration and 60 ns of NPT MD production. The pressure and temperature were maintained at 1 bar and 310 K, respectively. Regarding metadynamics, collective variables that varied during the simulation were first selected to calculate the potential mean force (PMF). The distance between the centroids of the amino acids of the protein and the peptide residues was chosen, particularly the C atoms of the backbone, from the binding amino acid residues in the intracellular loops of the receptor and all residues in the peptide. The original distance between the centroids, resulting from the MD-optimized coordinates of the docked structure, was set as the lower limit. During the metadynamics simulation, the peptide was forced in the direction of the intracellular water layer, parallel to the z-axis, and perpendicular to the plasma membrane. The frequency of the trajectory was 10,000 ps; the lower and upper wall constants and the width were 120.0, 180.0, and 0.1, respectively. The hill weight, width, and frequency were set to 0.1 kcal/mol, 1.0 Å, and 100 ps, respectively. The bias temperature was set at 1550 K. The 40 ns simulation used an integration step of 1 fs. Origin 8 (OriginLab Corporation, Northampton, MA, USA) was used for descriptive statistical analysis of the PMF and the generation of associated figures. Trajectory analysis was performed in USCF Chimera [28] and protein–peptide residue interaction network analysis in Cytoscape 3.10.1 [29] using structureViz2 plugin.

### 2.6. Fusion Constructs, Cell Culture, and Transfection

Fusion constructs C-terminal Renilla luciferase 8 (Rluc8)-tagged HAβ_2_AR (β_2_AR/Rluc8) and N-terminal GFP^2^-tagged β-arrestin 2 mutant (GFP2/β-arr2 R393E, R395E) were as previously described [30,31,32]. Human embryonal kidney 293 (HEK-293) cells (European Collection of Animal Cell Cultures, Salisbury, UK) were routinely maintained according to established protocols [30,33] and transiently transfected with Lipofectamine^®^-Plus™ reagent. Total luminescence and fluorescence were measured to monitor the expression of Rluc8- and GFP^2^-tagged constructs, respectively [34]. Trypan blue exclusion assay was used to monitor cell viability immediately after treatments. An aliquot (100 μL) of the cell suspension was diluted 1:1 (*v*/*v*) with 0.4% trypan blue solution (Gibco) and cells were counted with a hemocytometer under light microscope. Cell viability (%) was calculated by dividing the number of live (unstained) cells by the total number of cells (live and dead (blue-stained) cells).

### 2.7. Confocal Microscopy

HEK-293 cells (2.5 × 10^5^ cells/dish) were plated in a 35 mm glass bottom dish (WillCo Wells B.V., Amsterdam, The Netherlands). After 48 h at ~90% confluence, cells were washed with Dulbecco’s Phosphate-Buffered Saline (DPBS) and incubated in the dark with FITC-P4 (20 µg/mL) in supplemented DPBS (Ca^2+^/Mg^2+^, 1 g/L glucose, and 36 mg/L sodium pyruvate; sDPBS) for 1 h at room temperature. CellMask™ Orange plasma membrane stain (5 µg/mL; Thermo Fisher Scientific) was added during the last 10 min. At the end of incubation, the FITC-P4 and CellMask™ Orange stains were washed off and replaced with sDPBS. The cells were then immediately examined using a Leica confocal microscope (Leica TCS NT, Heidelberg, Germany). The 488 and 543 nm excitation laser lines were used to excite FITC-P4 and CellMask™ Orange stain, respectively. Sequential images were acquired at a resolution of 1024 × 1024 pixels. These images were presented using Adobe Creative Cloud version 6.1.0.587.7.

### 2.8. cAMP Assay

To examine the ability of P4 to interfere with isoproterenol-induced cAMP accumulation, the HitHunter^®^ cAMP assay (DiscoveRx Corporation Ltd., Fremont, CA, USA) was utilized and performed as previously described [33]. Briley HEK-293 cells were transiently transfected with 3 µg β_2_AR cDNA/75-cm^2^ flask. After 48 h, cells were resuspended in sDPBS with 0.5 mM 3-isobutyl-1-methylxanthine (IBMX) and plated at a density of 5 × 10^4^ cells/well in white 96-well plates (Packard BioScience, Meriden, CT, USA). Cells were treated for 60 min at room temperature with increasing concentrations of isoproterenol diluted in sDPBS (10^−12^ to 10^−5^ M) in the absence or presence of P4 (50 μM, final concentration) or with increasing concentrations of P4 (10^−9^ to 10^−4^ M). Subsequently, cAMP reagents were added, and the total luminescence was measured after 6 h in a TriStar2 S LB 942 microplate reader (Berthold Technologies, Bad Wildbad, Germany) at 1 s/well. The results were presented, and the EC_50_ values (nM ± SEM) were determined using a sigmoidal dose–response curve fit (GraphPad Prism 10.1.2, San Diego, CA, USA).

### 2.9. BRET^2^-Based β-Arrestin 2 Recruitment Assay

A previously described BRET^2^-based βarr2 recruitment assay [17,30,32,34] was employed. HEK-293 cells grown in a 75 cm^2^ flask were transiently co-transfected with β_2_AR/Rluc8 (0.1 μg) along with the GFP^2^/β-arr2 R393E, R395E mutant (4.9 μg). After 48 h, cells resuspended in sDPBS were plated onto 96-well white microplates at a 1 × 10^5^ cells/well (Packard BioScience, Meriden, CT, USA) density and treated for 60 min at room temperature with increasing concentrations of isoproterenol diluted in sDPBS (10^−12^ to 10^−5^ M) in the absence or presence of P4 (50 μM, final concentration) or with increasing concentrations of P4 (10^−9^ to 10^−4^ M). Subsequently, coelenterazine 400a was injected (final concentration of 5 μM), and luminescence signals at 410 nm and 515 nm were recorded using the TriStar2 S LB 942 microplate reader. The BRET^2^ signal represented a 515/410 ratio and was reported in milliBRET units (mBU): BRET^2^ ratio × 1000. The results were presented, and the EC_50_ values (nM ± SEM) were determined using a sigmoidal dose–response curve fit (GraphPad Prism 10.1.2, San Diego, CA, USA).

### 2.10. Microscale Thermophoresis (MST)

An estimated preliminary binding affinity (Kd) of ~15 µM for the β_2_AR:P4 interaction was determined by MST in a previous study [17].

## 3. Results

### 3.1. Computational Design and Modeling

**Figure 1:**
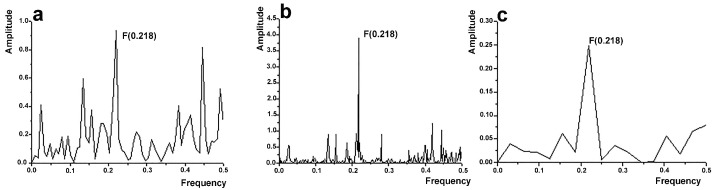
ISM bioinformatics analysis. (**a**) Informational spectrum (IS) of Nb71, (**b**) cross-spectrum (CS) of β_2_AR and Nb71, and (**c**) IS of P4 with the characteristic peak at F(0.218).

The ISM was initially used to design NDPs related to the β_2_AR by virtual spectroscopy. Sequences of conformationally selective Nbs favoring the agonist-bound active conformation of β_2_AR were analyzed [14,35], and the results were used to design peptide mimetics of Nb71, which preferentially binds to active, agonist-occupied β_2_AR [14]. The IS of Nb71, the cross-spectrum (CS) of Nb71 and β_2_AR, and the IS of the derived peptide mimetic are shown in Figure 1. Individual spectra of Nb71 (Figure 1a) and the CS of Nb71 and β_2_AR (Figure 1b) show that they have common frequency components, specifically F(0.218), indicating an interaction. Subsequently, the domains contributing to F(0.218) in the IS of Nb71 were identified using computer-assisted scanning. The peptide within CDR2, designated P4, consisting of 25 amino acids (AITTGGNTYYANSVKGRFTISRDNA), was identified. As shown in the IS, P4 shares informational properties, F(0.218), with Nb71 (Figure 1a,c), which supports the presence of an interaction with β_2_AR.

**Figure 2:**
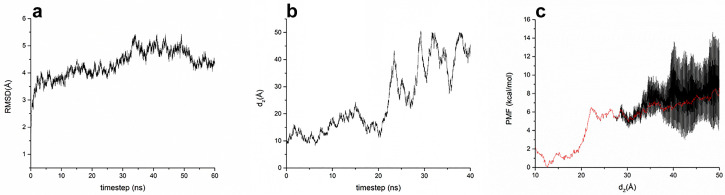
MD and metadynamics simulations. (**a**) RMSD plot of β_2_AR backbone atoms during the production simulation phase; (**b**) metadynamics trajectory plot of z-axis projection of the distance between the centroids, P4, and intracellular binding site amino acids of β_2_AR; (**c**) PMF profile of the unbinding β_2_AR-P4 event.

The β_2_AR:P4 complex was characterized via molecular docking of peptides, MD, and metadynamics simulations (Figure 2). A protocol similar to that used in a previous study [17] was used to select the docked peptides. The prepared β_2_AR:P4 complex was subjected to MD simulations. During the 60 ns of the production phase, the β_2_AR:P4 complex was stable, and the RMSD plot of the complex exhibited a convergence of the system (Figure 2a). Compared to the crystal structures of β_2_AR in complex with Nb80 (PDB 3P0G) and Gs protein (PDB 3SN6), the β_2_AR:P4 complex shares several key interacting residues after 60 ns of MD production (Table S1a). Of note, the interaction with β_2_AR residues Arg131 (part of the conserved DRY motif) and Ile135 was common to all three β_2_AR complexes (Table S1b). This can therefore be seen as additional validation of a size-independent peptide functionality. Furthermore, the interaction network determined from the trajectory confirms our assumptions that many interactions are formed and broken during the simulation due to peptide flexibility. There are long- and short-lived interactions, with the long-lived ones generally falling into the category of electrostatic interactions and contributing to the overall stability of the β_2_AR:P4 complex (Figure S1).

Subsequently, well-tempered metadynamics simulations of the β_2_AR:P4 complex were performed to assess the binding free energy between β_2_AR and P4. Analogous to a previous study [17], the carbon atoms were chosen from the backbone of the following residues: Arg41, Thr44, Arg109, Ile113, Pro116, Phe117, Gln120, Tyr197, Val200, Ala204, Lys207, Glu208, Ala211, Thr214, Leu215, Ile218, Tyr266, Arg268, Ser269, and Ile274. The centroid of β_2_AR was determined from the selected amino acid residues; additionally, all residues of P4 were determined from the centroid of the NDP. The initial distance between the two centroids was 10 Å, which gradually increased to 50 Å. P4 moved along the z-axis of the system into the intracellular water layer. The projection of the distance along the z-axis between β_2_AR and P4 during metadynamics simulation is shown in Figure 2b. Based on the PMF energy profile (Figure 2c), PMF energy initially increased to ~6 kcal/mol until the distance reached ~22 Å in the initial bound conformation. This energy change was due to the gradual decay of unbound protein–peptide interactions and the conformational energy of the peptide. A cumulative plot of the collected PMF outputs at timesteps of 30–40 ns is shown in Figure 2c, with a collection frequency of 10 ps. The process produced a very noisy PMF output after a dZ value of ~27 Å. According to the z-axis projection of the distance plot (Figure 2b), the non-binding protein–peptide interactions break down, and the peptide is pushed further into the intracellular water layer at that point. This event occurred after a time step of approximately 22 ns; additionally, the peptide was subjected to motion in the water layer under the influence of the applied force until the end of the metadynamics simulations. The wide PMF fluctuations were mainly due to changes in the conformational energy of the peptide. The peptide undergoes slow conformational changes, which may lead to free energies that are not completely converged and result in the fine structure of the PMF. The plots were difficult to interpret due to high deviations and contradictory observations (for example, the PMF values were higher in the water layer than those of the reaction barrier, at approximately 22 Å). Therefore, the binding free energies were assessed by averaging the PMF series. The red curve in Figure 2c represents the average of multiple PMF curves. Averaging several calculated PMF curves is valid because it improves the convergence of the binding free energy. This approach is routinely used in computational enzymology [36].

The median value of the average PMF output of multiple curves was calculated to estimate the binding free energy between 30 and 50 Å. The PMF difference was 6.78 kcal/mol, resulting in the following estimated binding free energy of the β_2_AR:P4 complex: ΔG = −6.78 kcal/mol or Ki = 16.5 μM at 310 K. The final result, including the standard error of the calculation (0.8 kcal/mol), was ΔG = (−6.8 ± 0.8) kcal/mol or Kd =15.9 μM. The corresponding experimental binding free energy was −6.55 kcal/mol.

The minimum conformational energy of P4 should be attained during 60 ns of simulation. Relying on one binding/unbinding event may be insufficient because achieving reasonable convergence of the PMF for such flexible ligands is challenging. However, the complete PMF output was followed in this study. Additionally, significant consensus with experimental results (estimated Kd value of ~15 µM in MST) was achieved. Therefore, this approach was retained, and the evaluation of the results was reliable. However, the agreement between the experimental and calculated binding free energies for such a flexible ligand could be a consequence of random error cancellation; therefore, the calculated mean force potential was appraised as semi-quantitative.

### 3.2. In Vitro Characterization of P4

**Figure 3:**
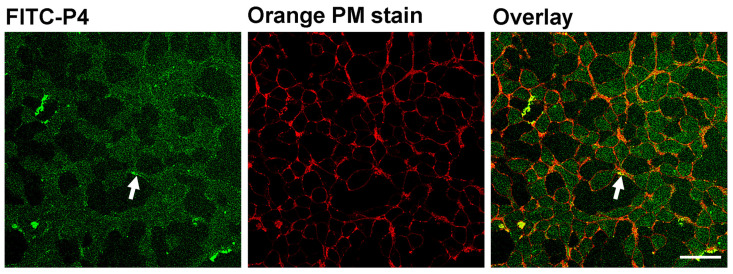
Microscopic evidence for FITC-labeled P4 entry into live HEK-293. Sequential confocal micrographs of live, nonpermeabilized HEK-293 cells after 1 h of incubation with FITC-labeled P4 (FITC-P4; green signal) and plasma membrane visualization with Orange plasma membrane stain (Orange PM stain; red signal). FITC-P4 translocates efficiently across the plasma membrane of live HEK-293 cells and exhibits a predominantly homogeneous cytoplasmic distribution and partial plasma membrane localization (white arrow), which is also shown by some degree of colocalization with the Orange plasma membrane marker (Overlay; yellow signal (white arrow)). Planapo oil objective 40×; scale bar, 10 μm.

**Figure 4:**
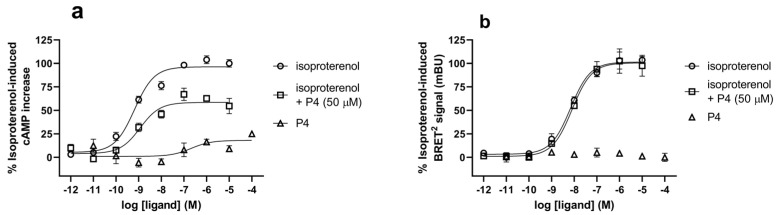
Effect of CDR2-NDP P4 on agonist-induced β_2_AR cAMP accumulation and interaction with β-arrestin 2. For the cAMP assay, (**a**) HEK-293 cells were transiently transfected with β_2_AR, and for the BRET^2^ β-arrestin recruitment assay, and (**b**) cells were transiently transfected with β_2_AR/Rluc8 along with the GFP^2^/β-arr2 R393E, R395E. In both assays, cells were treated with increasing concentrations of isoproterenol (10^−12^ to 10^−5^ M) in the absence or presence of P4 (50 μM, final concentration) or with increasing concentrations of P4 (10^−9^ to 10^−4^ M). Data (means ± S.E.) of three independent experiments, each performed in triplicate, are presented as a percentage of the maximum response induced by isoproterenol and plotted using a sigmoidal dose–response curve fit (GraphPad Prism 10.1.2).

The in vitro characterization of P4 was performed with regard to its ability to cross the plasma membrane and interfere with agonist-induced β_2_AR Gαs-mediated signaling and its interaction with β-arrestin 2 (Figure 3 and Figure 4). To verify the entrance of P4 into HEK-293 cells, FITC-labeled P4 (FITC-P4) was used. As shown in Figure 3, FITC-P4 was efficiently translocated through the plasma membrane and entered the HEK-293 cells. After 1 h of incubation, FITC-P4 displayed limited plasma membrane localization (as shown by some degree of colocalization with the Orange plasma membrane marker) and a predominantly homogeneous distribution throughout the cytoplasm.

The effect of P4 on the isoproterenol-induced interaction of β_2_AR with Gαs and β-arrestin 2 was assessed (Figure 4) because Nb71 has been demonstrated as a potent inhibitor of agonist-induced β_2_AR Gαs-mediated signaling and the interaction with β-arrestin 2 [14]. The level of cAMP was assessed in β_2_AR-transfected HEK-293 cells (Figure 4a). In β_2_AR-transfected HEK-293 cells, the agonist isoproterenol induced a dose-dependent increase in cAMP formation. This event occurred due to coupling with the stimulatory Gαs protein and subsequent adenylyl cyclase activation. The EC_50_ of isoproterenol was 0.86 ± 0.10 nM. Upon concomitant treatment with P4 (50 µM; final concentration), the maximal isoproterenol-induced cAMP level was reduced by approximately 40%, and isoproterenol potency also decreased by up to 20-fold (EC_50_ = 15.45 ± 8.0 nM vs. 0.86 ± 0.10 nM). Stimulation with P4 alone also increased cAMP formation, although its efficacy and potency were substantially lower than those with isoproterenol. In the absence of isoproterenol, P4 induced a limited (less than 20%) increase in cAMP; additionally, the estimated EC_50_ (209.5 ± 10.7 nM) shifted by >200-fold to the right, indicating that the interaction between P4 and the unstimulated β_2_AR was low in affinity. The trypan blue exclusion test also showed that the viability of the cells was not affected by a 1 h treatment with 50 µM P4, as both the control and P4-treated cells showed a viability of over 98%.

In contrast, P4 was inefficient at interfering with isoproterenol-induced β_2_AR/β-arrestin 2 interaction or β-arrestin 2 recruitment in the BRET^2^-based β-arrestin 2 recruitment assay (Figure 4b). BRET EC_50_ values (22.27 ± 7.79 and 25.05 ± 9.73 nM) for isoproterenol are similar in the absence and presence of M P4, respectively.

## 4. Discussion

The main goals of Nb downsizing are lower production costs, improved ability to cross the plasma membrane, and improved intracellular accessibility [15]. One possible approach is to prepare low-molecular-weight peptides, such as NDPs, based on their CDR sequences [2,15]. This study focused on the Nbs that are known to interact with agonist-activated β_2_AR [12,14]. The aim of this study was to computationally design CDR2-NDPs that mimic the biological functions of the Nb from which they were derived. In general, epitope recognition mainly depends on CDR3, whereas CDR1 and CDR2 were only assumed to establish additional interactions [2,12]. NDPs based on CDR3 of Nbs that stabilize the active state of β_2_AR were previously reported [16,17]. Bioinformatic ISM analysis identified the CDR2-NDP P4 that shared informational properties with Nb71 [14], supporting the interaction between the agonist-bound β_2_AR and P4. Molecular simulations of the β_2_AR:P4 complex yielded the following estimated binding free energy: ΔG = −6.78 kcal/mol or Ki = 16.5 μM. This corroborated the preliminarily determined Kd value of ~15 µM in MST. A recent computational study using the binding free-energy perturbation method reported unprecedented performance in classifying ligands as agonists or antagonists for various receptors, including GPCRs [37]. The estimated affinity of CDR2-NDP P4 was similar to a previously characterized Nb71 CDR3-NDP P3; therefore, the β_2_AR:P3 complex exhibited a −ΔG of 7.23 ± 0.04 kcal/mol or a Ki of 7.9 ± 0.5 μM [17]. The binding free-energy simulations involving flexible peptides are more demanding than those involving rigid ligands.

In vitro characterization of CDR2-NDP P4 demonstrated that it could effectively cross the plasma membrane of HEK-293 cells. This result corroborated the findings of Martin et al. [16], which showed that the entry of CDR3-derived peptide mimetics into HeLa cells was efficient. Additionally, these results suggest that P4 (consisting of 25 amino acid residues) behaves like cell-penetrating peptides, which typically comprise less than 30 amino acids and can cross the plasma membrane through energy-independent pathways. As discussed in a recent review, cell-penetrating peptides were already introduced in the GPCR field to target GPCR protein–protein interactions [38].

Nbs that stabilize the active state of GPCR bind to its intracellular domain in a cavity otherwise occupied by the α-subunit of G protein or arrestin [39]. Therefore, the effect of CDR2-NDP P4 on agonist-induced β_2_AR cAMP accumulation and interaction with β-arrestin 2 was assessed. The EC_50_ value of 0.86 ± 0.10 nM for β_2_AR-isoproterenol-induced cAMP accumulation in HEK-293 cells corresponded with previously published values (0.56 nM) [40]. P4 alone also induced a modest increase in cAMP without detectable β-arrestin recruitment, which may indicate its weak Gαs-biased agonism. Similarly to the development of biased allosteric modulators for β_2_AR, which aim to attenuate the side effects associated with the use of long-acting β_2_AR agonists by avoiding β-arrestin-mediated responses [41], Nbs/NDPs that act as Gαs-biased ligands for β_2_AR may represent a potential avenue for the development of prospective GPCR-targeted asthma therapeutics. Concomitant treatment with 50 µM P4 decreased the maximal isoproterenol-induced cAMP level by approximately 40% and reduced the potency of isoproterenol by up to 20-fold. Inhibition of isoproterenol-induced cAMP production by 20–30% has been reported for peptide mimetics of the Nb80 CDR3 [16]; this was also observed with Nb71 CDR3-NDP P3 (Supplementary Figure S2). Synthetic active-state specific β_2_AR Nbs were also shown to diminish adrenaline-mediated β_2_AR cAMP signaling by 45% [35]. Despite employing different methodologies for Nb/NDP generation (computational methods for P3 and P4, structural mimicry in the study by Martin et al., 2017 [16], and in vitro yeast surface display platform in the case of synthetic Nbs [35]), they all displayed moderate inhibitory effects on agonist-induced cAMP production, reaching up to 40–45%. In the case of CDR2-/CDR3 NDPs this could be attributed to their relatively low binding affinity owing to shorter lengths and smaller interaction interfaces than Nb80 and Nb71 [14].

In support of our computational and experimental data, CDR2-NDP P4 showed a similar interaction pattern to the C-terminal 14-amino acid peptide of Gαs [42]. Analogous to the C-terminal Gαs peptide interacting with the highly conserved DRY motif of β_2_AR, Lys15 of P4 interacts with Arg131 and Thr68 (Figure S1). In addition, Val14 interacts with Tyr141 and other nearby residues (Ala134, Pro138, and Gln142). However, the binding affinity and ability to modulate GPCR signaling do not always correlate as a synthetic active-state specific β_2_AR Nb.c203 with a lower binding affinity (151 nM) exhibited the highest effectiveness in cAMP assay [35]. A significant reduction in binding affinity/potency was generally reported when Nbs were downsized based on their CDRs [15]; however, binding and functional characteristics were preserved in the CDR3-derived 25-mer peptide directed against the vascular endothelial growth factor [18]. Mannes et al. [43] also reported the successful generation of G-protein-derived peptide mimetics using the α5-helix of Gαs instead of Nb CDR as a template (Gs-derived peptide mimetics). This study also highlights the necessity of stabilizing the secondary structure of peptide mimetics to enhance their receptor-binding affinity using various chemical stapling strategies and point mutations. The resulting 19-amino-acid-long peptide mimetic effectively stabilized the active state of the β_2_AR and dopamine D1 receptors with an impressive IC_50_ in the low nanomolar range [43]. Data obtained from CDR2- and CDR3-NDPs of Nb71 (P4 (this study) and P3 [17], respectively) suggest that, unlike Nb71 [14], they are inefficient at interfering with the receptor/β-arrestin 2 interaction. Given their short lengths (17 amino acid residues for P3 and 25 for P4), the receptor can be assumed to accommodate NDP and β-arrestin, considering that β-arrestins form larger interfaces with receptors than G-protein (reviewed in [44]). This assumption aligns with previous reports on the existence of megaplexes consisting of GPCR, β-arrestin bound only to the phosphorylated C-terminal tail, and the heterotrimeric G-protein [45]. Stable megaplexes were previously proposed only for class B GPCRs, which form strong interactions with the C-terminal tail, whereas in class A GPCRs, such as β_2_AR, their formation is limited [45,46]. In binding experiments of in vitro-translated β-arrestins to the C-terminal tail and the third intracellular loop of β_2_AR, β-arrestin 2 exhibited higher affinity for the C-terminal tail than for the third intracellular loop [47]. Furthermore, in a BRET^2^-based recruitment assay, a previously described β-arrestin 2 R393E, R395E mutant was used. This mutant was unable to interact with the components of clathrin-coated vesicles [30], resulting in class A GPCR behaving like class B GPCR and possibly favoring the “tail” interaction [43]. To the best of our knowledge, no previous studies described the ability of CDR3-NDPs or Gs-derived peptide mimetics to interfere with the agonist-induced β_2_AR interaction with β-arrestins. Considering that the β_2_AR:Gs complex interface is contiguous, whereas the interface between arrestin and receptor is interrupted and patchy (reviewed in [44]), one could speculate that NDPs that can effectively disrupt this interaction should be larger or targeted against a specific GPCR–β-arrestin interface.

## 5. Conclusions

In this study, CDR2-NDP P4 was computationally designed and characterized. It was shown to preferentially interact with agonist-activated β_2_AR and has the ability to interfere with the agonist-induced interaction of β_2_AR with Gαs without affecting the β-arrestin-2 interaction. Its binding affinity and ability to interfere with the agonist-induced interaction of β_2_AR with Gαs are similar to those of CDR3-derived peptide mimetics. The β_2_AR:P4 complex also shares key interacting residues with the β_2_AR:Nb80 and β_2_AR:Gs complexes, suggesting that additional residues outside the CDR3 are important for effective interference with Gαs interactions, and our data provide evidence for the involvement of residues located in the CDR2. In analogy with the development of Gs-derived peptide mimetics, CDR-derived peptide mimetics are promising candidates for further chemical modification and optimization.

## Data Availability

All data generated or analyzed during this study are included in this published article and its Supplementary Information Files.

## Supplementary Materials

The following supporting information can be downloaded at: https://www.mdpi.com/article/10.3390/biom14040423/s1, Table S1: (a) List of interacting residues between β_2_AR and (i) CDR2-NDP P4 after 60 ns of MD simulation, (ii) Nb80 (PDB P0G), and (iii) Gs protein (PDB 3SN6). The common interacting residues in β_2_AR for all three structures are highlighted in red. (b) Summary of common interaction residues for all three β_2_AR complex structures; Figure S1: The amino acid interaction network between β_2_AR (chain A, yellow) and CDR2-NDP P4 (chain B, cyan); Figure S2: Effect of CDR3-NDP P3 on agonist-induced β_2_AR cAMP accumulation in HEK-293 cells.
