# Supplementary material for: Computational Modeling and Characterization of Peptides Derived from Nanobody Complementary-Determining Region 2 (CDR2) Targeting Active-State Conformation of the β2-Adrenergic Receptor (β2AR)
Source: Biomolecules. 2024 Mar 30;14(4):423. doi: 10.3390/biom14040423 (PMC11048008; doi:10.3390/biom14040423)
Supplement: Supplementary file 1 [file biomolecules-14-00423-s001.zip › Figure S1.pdf]

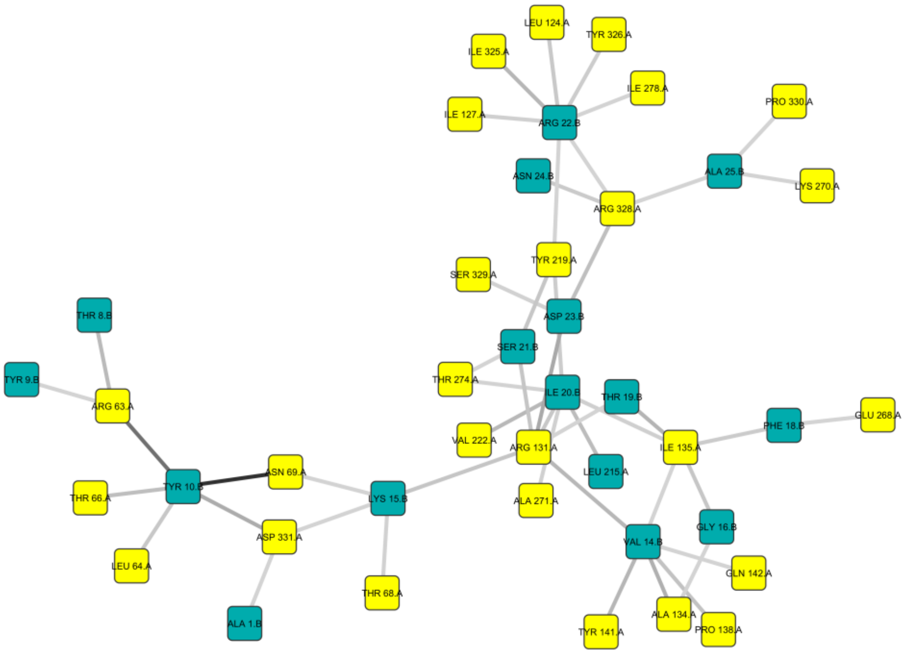

Figure S1. The amino acid interaction network between β_2AR (chain A, yellow) and CDR2-NDP P4 (chain B, cyan). Light gray lines: short lived interactions; dark grey lines: long lived interactions. Light gray lines: short lived interactions; dark grey lines: long lived interactions.
