# Supplementary material for: Computational Modeling and Characterization of Peptides Derived from Nanobody Complementary-Determining Region 2 (CDR2) Targeting Active-State Conformation of the β2-Adrenergic Receptor (β2AR)
Source: Biomolecules. 2024 Mar 30;14(4):423. doi: 10.3390/biom14040423 (PMC11048008; doi:10.3390/biom14040423)
Supplement: Supplementary file 1 [file biomolecules-14-00423-s001.zip › Figure S2.pdf]

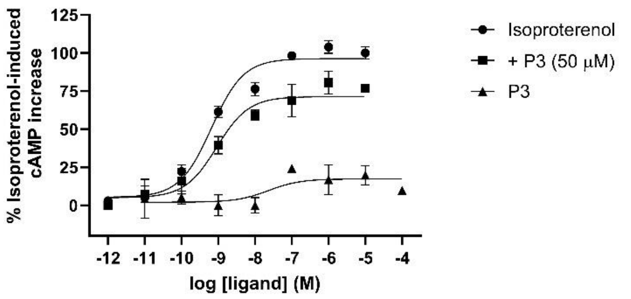

Figure S2. Effect of CDR3-NDP P3 on agonist-induced β2AR cAMP accumulation in HEK-293 cells. HEK-293 cells transiently transfected with β2AR were treated with increasing concentrations of isoproterenol (10^{-12} to 10^{-5} M) in the absence or presence of P3 (50 μM, final concentration) or with increasing concentrations of P3 (10^{-9} to 10^{-4} M). Data (means ± S.E.) of three independent experiments, each performed in triplicate, are presented as a percentage of the maximum response induced by isoproterenol and plotted using a sigmoidal dose-response curve fit (GraphPad Prism 10.1.2).
