# Supplementary material for: Computational Modeling and Characterization of Peptides Derived from Nanobody Complementary-Determining Region 2 (CDR2) Targeting Active-State Conformation of the β2-Adrenergic Receptor (β2AR)
Source: Biomolecules. 2024 Mar 30;14(4):423. doi: 10.3390/biom14040423 (PMC11048008; doi:10.3390/biom14040423)
Supplement: Supplementary file 1 [file biomolecules-14-00423-s001.zip › Table S1.pdf]

Table S1: **(a)** List of interacting residues between β2AR and i) CDR2-NDP P4 after 60ns of MD simulation, ii) Nb80 (PDB P0G) and iii) Gs protein (PDB 3SN6). The common interacting residues in β2AR for all three structures are highlighted in red. **(b)** Summary of common interaction residues for all three β2AR – complex structures.

a

**β2AR**	**CDR2-NDP P4**	**Distance between donor and acceptor atom (Å)**	**Intermolecular interactions category**	**Appearing and common residues in β2AR**
Asp331	Lys15	1.7014	Hydrogen Bond; Electrostatic	Asp331
Asn69	Tyr10	1.8536	Hydrogen Bond	Asn69
Arg343	Ile2	2.0403	Hydrogen Bond	Arg343
Asp331	Thr4	1.7517	Hydrogen Bond	Arg328
Arg328	Ala25	3.0077	Hydrogen Bond	Arg131
Asp331	Lys15	3.0632	Hydrogen Bond	Ala134
Arg328	Asn24	2.4460	Hydrogen Bond	Ile135
Arg131	Val14	5.4249	Hydrophobic	
Arg131	Ile20	4.8244	Hydrophobic	
Ala134	Val14	3.9120	Hydrophobic	
Ile135	Val14	5.3470	Hydrophobic	
Ile135	Phe18	5.0223	Hydrophobic	
**3P0G**	**Nb80**			
Arg131	Glu106	4.5550	Electrostatic	Arg131
Arg131	Val103	2.779	Hydrogen Bond	Ala226
Ala226	Ser30	3.7618	Hydrogen Bond	Ile135
Ile135	His52	2.9527	Hydrogen Bond	Arg328
Arg328	Tyr105	4.4218	Hydrophobic	Val222
Arg131	Leu104	5.4517	Hydrophobic	Ile278
Val222	Val103	5.4757	Hydrophobic	Leu275
Ala226	Ile31	5.1657	Hydrophobic	Phe139
Ile278	Leu104	5.2701	Hydrophobic	Tyr326
Leu275	Val103	4.8830	Hydrophobic	Ala271
Phe139	Ala50	4.7444	Hydrophobic	Pro138
Tyr326	Leu104	5.1916	Hydrophobic	Pro330
Ala226	Phe29	5.2954	Hydrophobic	
Ala271	Phe29	4.4089	Hydrophobic	
Pro138	His52	4.6784	Hydrophobic	
Ile135	Tyr100	5.3995	Hydrophobic	
Pro330	Tyr105	5.2751	Hydrophobic	
**3SN6**	**Gαs**			
Lys232	Asp381	3.0736	Hydrogen Bond; Electrostatic	Lys232
Glu225	Arg380	4.4865	Electrostatic	Glu225
Arg228	Asp381	4.3197	Electrostatic	Arg228
Arg239	Asp343	4.6927	Electrostatic	Arg239
Thr136	Arg380	3.0563	Hydrogen Bond	Thr136

Ile135	Gln384	2.9327	Hydrogen Bond	Ile135
Glu225	Gln384	3.0383	Hydrogen Bond	Ala134
Ala134	His387	3.1922	Hydrogen Bond	Gln229
Gln229	Gln384	2.7634	Hydrogen Bond	Arg131
Arg131	Tyr391	4.6482	Electrostatic	Phe139
Ala134	His387	3.8565	Hydrophobic	Tyr141
Phe139	His41	5.1673	Hydrophobic	Leu275
Phe139	Phe376	4.7377	Hydrophobic	Leu230
Tyr141	His387	5.0636	Hydrophobic	Pro138
Ile135	Leu388	4.9550	Hydrophobic	Ala226
Leu275	Leu393	5.2230	Hydrophobic	Ile233
Leu230	Leu394	5.3018	Hydrophobic	
Pro138	Ile383	4.6206	Hydrophobic	
Ala226	Leu388	4.4277	Hydrophobic	
Ile233	Tyr358	4.9347	Hydrophobic	
Arg131	Tyr391	4.3581	Hydrophobic	
Phe139	Val217	4.43179	Hydrophobic	
Phe139	Arg380	4.3352	Hydrophobic	

b

**β_2AR residue**	**Interaction with CDR2-NDP P4**	**Interaction with Nb80 (PDB P0G)**	**Interaction with Gs protein (PDB 3SN6)**
Arg131	yes	yes	yes
Ile135	yes	yes	yes
Arg328	yes	yes	no
Ala134	yes	no	yes
Ala226	no	yes	yes
Leu275	no	yes	yes
Phe139	no	yes	yes
Pro138	no	yes	yes
